# Molecular Engineering of Curcumin, an Active Constituent of *Curcuma longa* L. (Turmeric) of the Family *Zingiberaceae* with Improved Antiproliferative Activity

**DOI:** 10.3390/plants10081559

**Published:** 2021-07-29

**Authors:** Amena Ali, Abuzer Ali, Abu Tahir, Md. Afroz Bakht, Salahuddin, Mohamed Jawed Ahsan

**Affiliations:** 1 Department of Pharmaceutical Chemistry, College of Pharmacy, Taif University, P.O. Box 11099, Taif 21944, Saudi Arabia; 2 Department of Pharmacognosy, College of Pharmacy, Taif University, P.O. Box 11099, Taif 21944, Saudi Arabia; abuali@tu.edu.sa; 3 Department of Pharmacology, Raghukul College of Pharmacy, Bhopal 462 003, India; aliabutahir2009@gmail.com; 4 Department of Chemistry, College of Science and Humanity Studies, Prince Sattam Bin Abdulaziz University, P.O. Box 83, Al-Kharj 11942, Saudi Arabia; bakhtpharm@gmail.com; 5 Department of Chemistry, Noida Institute of Technology (Pharmacy Institute), Knowledge Park-2, Greater Noida 201 306, India; sallu_05@yahoo.co.in; 6 Department of Pharmaceutical Chemistry, Maharishi Arvind College of Pharmacy, Ambabari Circle, Jaipur 302 039, India

**Keywords:** antiproliferative agents, anti-EGFR, curcumin analogues, molecular docking, cancer cell lines

## Abstract

Cancer is the world’s second leading cause of death, accounting for nearly 10 million deaths and 19.3 million new cases in 2020. Curcumin analogs are gaining popularity as anticancer agents currently. We reported herein the isolation, molecular engineering, molecular docking, antiproliferative, and anti-epidermal growth factor receptor (anti-EGFR) activities of curcumin analogs. Three curcumin analogs were prepared and docked against the epidermal growth factor receptor (EGFR), revealing efficient binding. Antiproliferative activity against 60 NCI cancer cell lines was assessed using National Cancer Institute (NCI US) protocols. The compound **3b**,**c** demonstrated promising antiproliferative activity in single dose (at 10 µM) as well as five dose (0.01, 0.10, 1.00, 10, and 100 µM). Compound **3c** inhibited leukemia cancer panel better than other cancer panels with growth inhibition of 50% (GI_50_) values ranging from 1.48 to 2.73 µM, and the most promising inhibition with GI_50_ of 1.25 µM was observed against leukemia cell line SR, while the least inhibition was found against non-small lung cancer cell line NCI-H226 with GI_50_ value of 7.29 µM. Compounds **3b**,**c** demonstrated superior antiproliferative activity than curcumin and gefitinib. In molecular docking, compound **3c** had the most significant interaction with four H-bonds and three π–π stacking, and compound **3c** was found to moderately inhibit EGFR. The curcumin analogs discovered in this study have the potential to accelerate the anticancer drug discovery program.

## 1. Introduction

Natural products (NPs) have been used to treat human ailments since the dawn of medicine. NPs have historically been a highly prolific source of new medicines since the time immemorial in most of the civilizations. NPs continue to offer a diverse set of structural templates for drug discovery and development, which was heightened in the 1970s and 1980s [1,2]. Many of today’s small molecule therapeutics can be traced back to NPs, which are anticipated to have provided or inspired the development of 50–70% of all agents currently in clinical use [3]. Studies focused on the isolation, structural elucidation, and biological activities of substances derived from NPs began around the end of the 18th century and introduction of column chromatography revolutionized an era of NP chemistry [4]. A number of anticancer drugs have been developed by chemically modifying plant NPs. Epothilone B, derived from *Sorangiumcellulosum*, has been chemically modified to Ixabepilone [5]. Camptothecin, an alkaloid derived from *Camptotheca acuminate*, has been modified into various anticancer agents including Karenitecin (BNP-1350), Diflomotecan (BN80915), and Gimatecanetc [6,7,8]. Tafluposide has been modified from Epipodophyllotoxin, an alkaloid derived from *Podophyllum peltatum* [9]. Acronycine is an alkaloid derived from *Acronychiabaueri* that has been modified to S23906-1 [10]. Combretastatin A-4 is an alkaloid derived from *Combretum caffrum* thathas been converted into anticancer agents such as Combretastatin A-4 phosphate, Combretastatin A-4 diphosphate and Ombrabulin (AVE8062) [11,12,13]. Terameprocol has been synthesized from 3′-*O*-methyl-nordihydroguaiaretic acid, a lignin isolated from *Larreadivaricatta* [14]. Daidzein, an isoflavone found in a variety of plants and herbs such as *Pueraria Mirifica*, soybeans, and soy products, has been converted to Triphendiol and Genistein [15,16].

Curcumin, one of the active constituents of *Curcuma longa* L. of the family (Zingiberaceae), has had its structure modified in order to produce biologically active compounds. The chemical modification improved not only the biological activities but also the stability, reducing rotational freedom and minimizing metal-chelation properties [17]. Curcumin analogues demonstrated broad spectrum activities as anticancer, anti-inflammatory, anti-HIV, anti-Alzheimer’s, antimicrobial, epidermal growth factor receptor (EGFR) inhibitors, and other biological activities as reported in the previous studies [18,19,20,21,22,23,24,25,26,27,28].

Cancer is one of the most dreadful diseases and the leading cause of death today, with nearly 10 million deaths and 19.3 million new cases expected in 2020 [29]. Chemotherapy is the most commonly used method of cancer treatment, but it is always associated with a number of toxic side effects. As a result, we became more reliant on nature, because active constituents of natural origin are assumed to be safe. In some studies, curcumin analogs were also discovered to be an inhibitor of EGFR, one of the primary targets of many anticancer drugs [18,28]. Furthermore, many of the cancer cell lines, including breast (MDA-MB-231, MCF7, HS 578T, BT-549, T-47D MDA-MB-468, and MDA-MB-468), colon (HCT-116, HT-29, COLO 205, HCT 116, HT29, and SW620), non-small cell lung (A549, NCI-H460, and NCI-H322), renal (786-O), melanoma (SKMEL28), ovarian (SKOV3), and prostate (PC3) cancer cell lines, have been found to express varying levels of EGFR [30,31,32,33,34,35,36]. Inspired by their impressive biological activities, we reported herein the molecular docking, antiproliferative potential, and anti-EGFR activity of some of the prepared curcumin analogs (**3a**,**b**). In silico molecular docking analysis was carried out against EGFR through the Schrodinger’s software. As molecular docking is now an integral part of computer-aided drug design (CADD), in which ligands with computed generated 3D structures are positioned into a receptor or biological target in a variety of orientations, conformations, and positions [37], it has numerous uses and applications in drug discovery, including structure–activity studies, lead optimization, and virtual screening to find potential leads [38]. Indeed, molecular docking has been used for more than three decades, and it has resulted in the discovery and development of a large number of new drugs [39].

## 2. Methods and Materials

### 2.1. Procurement of Chemicals and Crude Turmeric

All of the chemicals were obtained from a local vendor. The standard curcumin was obtained from Konark Herbals in India, and the remaining chemicals and reagents were obtained from Merck Chemicals, SD Fine, and CDH. All of the chemicals were either analytical or synthetic grades in nature. The dry turmeric was purchased from a local market and ground into powder.

### 2.2. Isolation of Curcumin

Eighty grams of ground turmeric was refluxed in 200 mL of dichloromethane for one hour with continuous stirring, followed by filtration under vacuum. The filtrate was concentrated under vacuum while the temperature was kept at 50 °C. The reddish yellow oily residue was triturated further with 80 mL of hexane to yield 2.47 g of solid residue. The resulting solid residue was dissolved in a small amount of dichloromethane/ methanol (99:1) and loaded into a column packed with silica gel. The column was eluted with the same mobile phase, and curucmin was isolated, collected, and analyzed using thin layer chromatography (TLC) with standard curcumin as a reference. For the isolation of curcumin the conventional method was used and with 80 g of curcumin powder nearly 244 mg of curcumin was isolated [40].

### 2.3. Preparation of Curcumin Analogues

**Figure 1:**
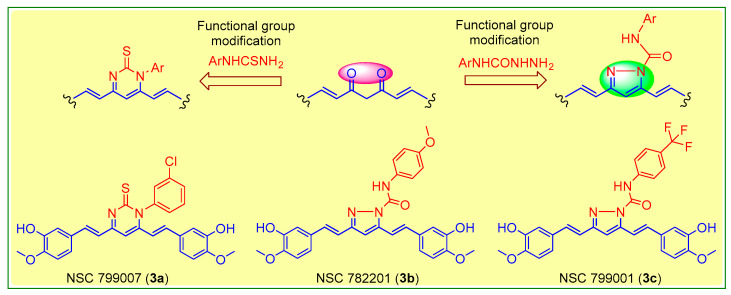
Molecular engineering of curcumin to pyrimidine thione (**3a**), and pyrazoles (**3b**,**c**) counterparts.

**Scheme 1:**
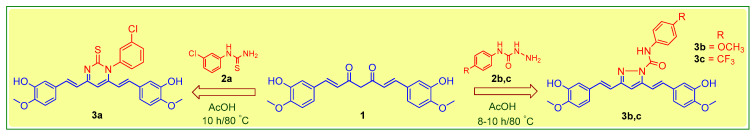
Molecular engineering for the synthesis of curcumin analogs (**3a**–**c**).

The isolated curcumin was used as a starting compound for the preparation of curcumin analogues via chemical modification of curcumin according to the methods described [18,19]. Figure 1 depicts the chemical modification curcumin and curcumin analogues. An equimolar amount of curcumin (**1**) (0.25 mmol; 92 mg) and 3-chlorophenyl thiourea (**2a**) (0.25 mmol; 46 mg) in glacial acetic acid in 50 mL round-bottom flask placed in sand bath was heated at 80 °C with continuous stirring on magnetic stirrer for 10 h to obtain the compound **3a**, while an equimolar amount of curcumin (**1**) (0.25 mmol; 92 mg) and *N*-(4-substitutedphenyl)hydrazine carboxamides (**2b**,**c**) (0.25 mmol) in glacial acetic acid in 50 mL round-bottom flask placed in sand bath was heated at 80 °C with continuous stirring for 8–10 h on magnetic stirrer to obtain the compound **3b**,**c**. The completion of reaction was monitored throughout by thin layer chromatography using mobile phase, *n*-hexane: ethylacetate (6:4). The excess of solvent was removed under vacuum distillation. Cooled the reaction mixture to room temperature and poured into the crushed ice to obtain the crude product, filtered, and dried. The crude products were further re-crystallization with ethanol to obtain the compounds, **3a**–**c**. The synthetic route followed is shown in Scheme 1.

### 2.4. Molecular Docking Studies

The docking was done against epidermal growth factor receptor (EGFR), which is one of the most appealing targets for many anticancer drugs. The protein data bank provided the EGFR (PDB:3W2R) X-ray crystal structure with a resolution of 2.05 Å; R-value 0.220 (observed) [41]. The ligands **3a**–**c** were saved as mol files, and docking was performed according to the protocol described by Sogabe et al. (2013) [42].

### 2.5. Antiproliferative Activity

The antiproliferative activity of tested compounds was done against five dozen (60) cancer cell lines derived from nine different panels. All of the compounds were evaluated for antiproliferative activity in a single dose at 10 µM. Growth percent (GP) and percent growth inhibition (% GI) were used to calculate antiproliferative activity. The tested compounds that demonstrated significant activity were subjected to a five-dose assay (0.01 to 100 µM). The antiproliferative results of five-dose assay were calculated as three dose related parameters like, 50% growth inhibition (GI_50_), 50% lethal concentration (LC_50_), and total growth inhibition TGI [43,44,45,46,47].

Human tumor cell lines were grown in RPMI 1640 medium containing 5% fetal bovine serum and 2 mM l-glutamine. For a typical screening experiment, cells were inoculated into 96-well microtiter plates in 100 µL at plating densities ranging from 5000 to 40,000 cells/well, depending on the doubling time of individual cell lines. Two plates of each cell line were fixed in situ with trichloroacetic acid (TCA) after 24 h to represent a cell population measurement for each cell line at the time of drug addition (Ti). The experimental drugs were frozen after being solubilized in dimethyl sulfoxide at 400-fold the desired final maximum test concentration. An aliquot of frozen concentrate was thawed and diluted to twice the desired final maximum test concentration with medium containing 50 µg/mL gentamicin at the time of drug addition. Additional four, 10-fold, or ½ log serial dilutions serial dilutions were performed to provide a total of five drug concentrations plus the control. Aliquots of 100 µL of these different drug dilutions were added to the appropriate microtiter wells already containing 100 µL of medium, resulting in the required final drug concentrations. Following drug addition, the plates are incubated for an additional 48 h at 37 °C, 5% CO_2_, 95% air, and 100% relative humidity. For adherent cells, the assay is terminated by the addition of cold TCA. Cells are fixed in situ by the gentle addition of 50 µL of cold 50% (*w*/*v*) TCA (final concentration, 10% TCA) and incubated for 60 min at 4 °C. The supernatant is discarded, and the plates are washed five times with tap water and air dried. Sulforhodamine B (SRB) solution (100 µL) at 0.4% (*w*/*v*) in 1% acetic acid was added to each well, and plates were incubated for 10 min at room temperature. After staining, unbound dye was removed by washing five times with 1% acetic acid and the plates were air dried. Bound stain was subsequently solubilized with 10 mM trizma base, and the absorbance was read on an automated plate reader at a wavelength of 515 nm. For suspension cells, the methodology was the same except that the assay was terminated by fixing settled cells at the bottom of the wells by gently adding 50 µL of 80% TCA (final concentration, 16% TCA).

### 2.6. Anti-EGFR Activity

According to the reported method, a spectrophotometric assay for EGFR tyrosine kinase inhibition (absorbance at λ_max_ 540 nm) was performed in human A431 cells, which express higher levels of EGFR protein [48].

## 3. Results

### 3.1. Molecular Docking Studies

**Figure 2:**
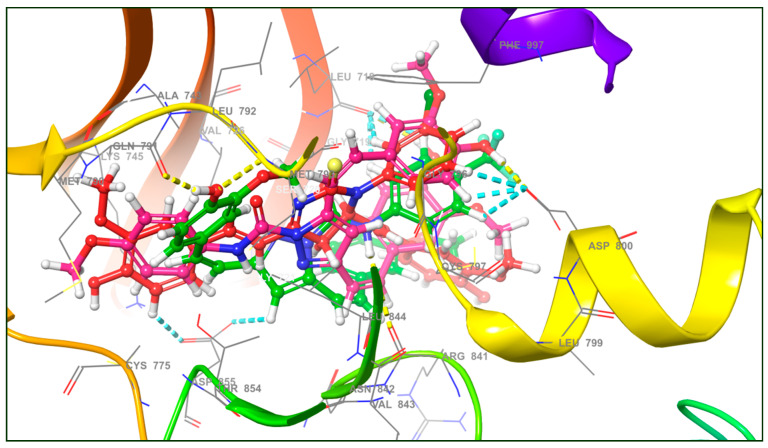
The binding mode of curcumin analogs (**3a**–**c**) and various types of interactions (yellow dotted line (**---**) showing H-bond and sky blue dotted line (**---**) showing π–π stacking) with amino acid residues in docking studies against the active site of EGFR.

**Figure 3:**
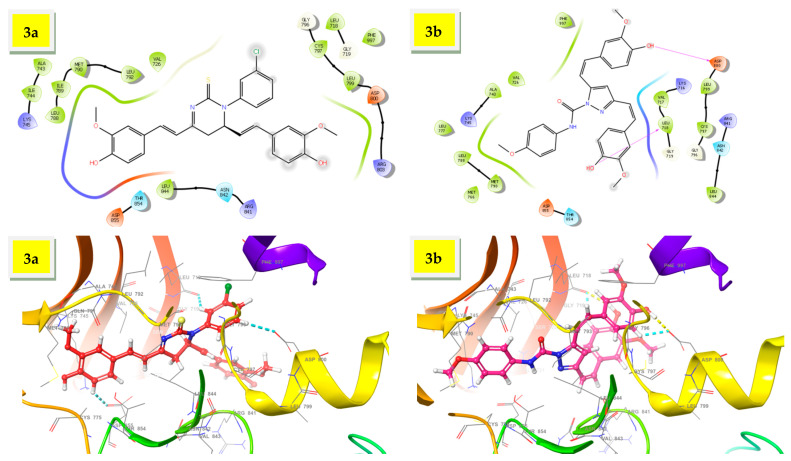
The 3D interaction of ligands, **3a**,**b** within the active site of EGFR.

**Figure 4:**
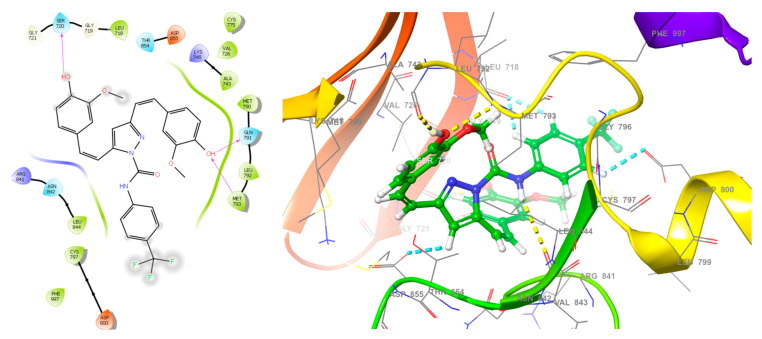
The 2D and 3D interactions of ligand **3c** within the active site of EGFR.

EGFR was chosen for docking studies because it is a potential target for many anticancer drugs, including gefitinib, erlotinib, cetuximab, and panitumumab [49]. Curcumin analogues have also been well studied as anti-EGFR agents [18,28]. The X-ray crystal structure of EGFR with PDB ID: 3W2R was retrieved from protein data bank [41]. The molecular docking score and types of interaction of curcumin analogs (**3a**–**c**) are given in Table 1 and binding mode of curcumin analogs are shown in Figure 2. Due to their large structure and high molecular weight, the curcumin analogs fit inside the active site of EGFR and showed various types of interaction including hydrophobic interaction, H-bond, and π–π stacking. The ligand **3a** displayed only π–π stacking interaction with the amino acids Asp855, Asp800, and Leu718, while ligand **3b** displayed two H-bond with the residues Leu718 and Asp800 and two π–π stacking with the residues Leu718 and Asp800 as showed in Figure 3. The ligand **3c** displayed significant interaction within the active site of EGFR having three H-bonds with the residues Met793, Gln791, and Ser720, while three π–π stacking with the residues Asp855, Asp800, and Leu718 as shown in Figure 4.

### 3.2. Antiproliferative Activity

**Figure 5:**
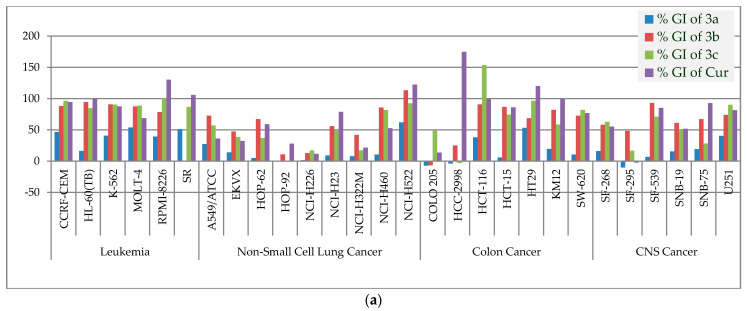

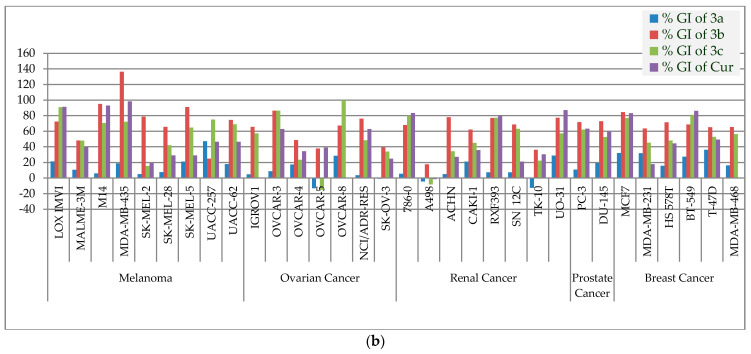
(**a**). The % GIs of curcumin analogs (**3a**–**c**) and curcumin (Cur) on leukemia, non-small cell lung, colon, and CNS cancer panels. (**b**). The % GIs of curcumin analogs (**3a**–**c**) and curcumin (Cur) on melanoma, ovarian, renal, prostate, and breast cancer panels.

The ligands **3a**–**c** demonstrated significant interactions (docking score: approximately−6.337 to 6.593 kcal/mol) in the molecular docking studies and were further tested for antiproliferative activity against five dozen (60) human cancer cell lines derived from nine different panels, including breast, colon, CNS, leukemia, melanoma, non-small cell lung, prostate, and renal cancer cell lines, according to National Cancer Institute (NCI US) protocols [43,44,45,46,47]. In a one-dose assay (Figures S2–S4), compound **3a** inhibited growth by more than 50% in three cancer cell lines—MOLT-4 (% GI = 53.78), HT29 (% GI = 53.43), and SR (% GI = 51.28), while the remaining cell lines (56 cell lines) inhibited growth by less than 50%. Compound **3b** was found to be lethal against two cell lines—NCI-H522 and MDA-MB-435, with percent GIs of 113.52 and 136.25, respectively, whereas compound **3c** had lethal effect against RPMI-8226 and HCT-116, with percent GIs of 100.21 and 153.67, respectively (shown in red bold font in the Table 2). The compound **3b** was found to be active against 32 cell lines, while the compound **3c** was found to be active against 25 cell lines with % GIs of ≥68% (shown in green bold font in the Table 2). The compound is supposed to be active if it inhibited the growth of cancer cell lines by ≥68 percent [50,51]. Compound **3b** demonstrated >50% GIs (and <68% GIs) against 13 cell lines, while compound **3c** demonstrated >50% GIs against 12 cell lines (shown in blue bold font in the Table 2). Compound **3b** demonstrated <50% GIs against 9 cell lines, while compound **3c** demonstrated <50% GIs against 17 cell lines (shown in black font in the Table 2). Curcumin was more antiproliferative than compound **3a**. Compounds **3b** demonstrated better inhibition than curcumin against 35 of the 56 cell lines in common, while compound **3c** displayed better inhibition than curcumin against 27 of the 56 cell lines in common. Figure 5a,b shows a comparison of antiproliferative activity. The results of mean % GIs on each panel was compared, and it was found that compound **3b** displayed better inhibition than curcumin against seven panels and better inhibition than gefitinib against six cell lines panels, while compound **3c** displayed better inhibition than curcumin against the four cell lines panels and better inhibition than gefitinib against five cell lines panels in one dose assay at 10 µM (Table 3). The order of antiproliferative activity of curcumin analogues was found to be **3b** > **3c** > **3a**. Compounds **3b**,**c** demonstrated promising antiproliferative activity in a single dose assay and thus qualified for further evaluation in a five-dose assay (0.01, 0.10, 1, 10, and 100 µM).

**Figure 6:**
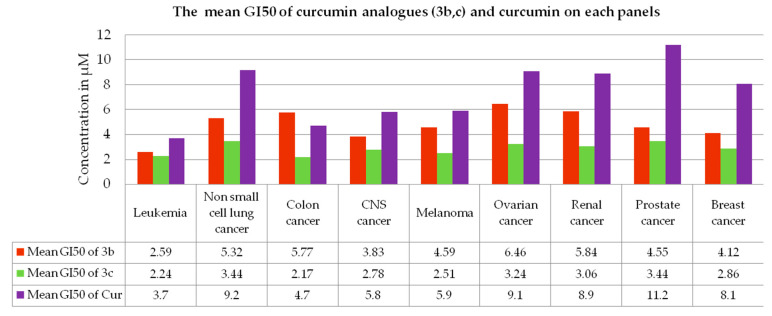
The mean GI_50_ of curcumin analogs (**3b**,**c**) and curcumin (Cur) on each panel of cancer cell lines in the five-dose assay.

**Figure 7:**
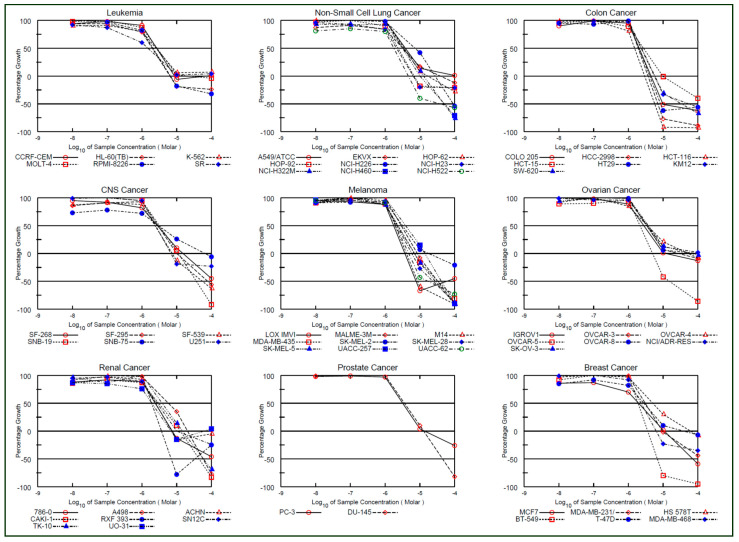
Antiproliferative profile of compound **3c** in terms of GP and Log_10_ molar concentration.

Compounds **3b**,**c**, which demonstrated promising antiproliferative activity against some of the cancer cell lines in a single dose assay with GIs of >68 (GP 32 percent), were chosen for further evaluation in a five-dose assay [50,51]. The antiproliferative activity of compounds (**3b**,**c**) was represented as a three-dose-related parameter: GI_50_, TGI, and LC_50_. The compound **3b** had GI_50_ values ranging from 1.25 to 18.8 µM, TGI values ranging between 6.69 and >100 µM and LC_50_ > 100 µM, whereas the compound **3c** had GI_50_ values ranging from 1.48 to 7.29 µM, TGI values ranging between 2.06 and >100 µM and LC_50_ > 100 µM. Compounds **3b**,**c** showed promising inhibitions against SR in leukemia cell lines’ panel, with GI_50_ values of 1.52 and 1.48, respectively, whereas the compounds **3b**,**c** showed promising inhibitions against NCI-H522 in non-small lung cancer cell lines‘ panel with GI_50_ values of 1.76 and 1.78, respectively. Likewise, compounds **3b**,**c** showed promising inhibitions against SF-539 in CNS cell lines’ panel, with GI_50_ values of 2.35 and 2.37 respectively, whereas the compounds **3b**,**c** showed promising inhibitions against UO-31 in renal cancer cell lines’ panel with GI_50_ values of 1.52 and 1.94, respectively. Compound **3b**,**c** showed promising inhibitions against BT-549 in breast cell lines’ panel with GI_50_ values of 2.49 and 1.96, respectively. Compounds **3b**,**c** showed promising inhibitions, respectively, against HCT-15 and HCT-116 in colon cancer cell lines’ panel with GI_50_ values of 2.98 and 1.51 respectively, whereas compounds **3b**,**c** showed promising inhibitions, respectively, against MDA-MB-435 and LOXIMVI in melanoma cell lines panel with GI_50_ values of 1.25 and 1.77 respectively. Compounds **3b**,**c** showed promising inhibitions respectively against OVCAR-3 and OVCAR-5 in ovarian cancer cell lines’ panel with GI_50_ values of 2.98 and 1.16, respectively, whereas compounds **3b**,**c** showed promising inhibitions, respectively, against DU-145 and PC-3 in prostate cancer cell lines’ panel with GI_50_ values of 3.99 and 3.21, respectively. Furthermore, the selectivity ration (SR) of compound **3b** ranging between 0.76 and 1.89, whereas the selectivity ration of compound **3b** ranging between0.82 and 1.31 indicated their non-selectivity towards the cancer cell line panels. Table 2 summarizes the results of the 5-dose assay. The mean GI_50_ of each panel was calculated and compared to that of curcumin. Except for the panel colon cancer by compound **3b**, both the curcumin analogs (**3b**,**c**) demonstrated more promising antiproliferative activity with better inhibition than curcumin as shown in Figure 6. The antiproliferative activity of compounds **3c** is shown in Figure 7, while antiproliferative activity of compound **3b** against nine panels of 60 NCI cancer cell lines in terms of GP and Log10 molar concentration are shown in Figure S1 (In Supplementary Materials). In a 5-dose assay, the overall antiproliferative activity was **3c** > **3b** > curcumin. Curcumin molecular engineering into semi-synthetic curcumin analogues (**3b**,**c**) resulted in increased antiproliferative activity.

### 3.3. Anti-EGFR Activity

The ligands (**3b**,**c**) with promising antiproliferative activity and displayed significant interaction within the active site of EGFR in molecular dockingwere then tested for anti-EGFR activity in human A431 cells, which express high levels of EGFR protein, using a spectrophotometric assay (absorbance at λ_max_ 540 nm). Both the compounds (**3b**,**c**) moderately inhibited the EGFR with IC_50_ values of 3.89 and 4.18 µM, respectively, while the standard drug, gefitinib inhibited EGFR with IC_50_ value of 0.017 µM.

## 4. Discussion

Turmeric has been promoted as a therapeutic herb in the majority of indigenous traditional systems, including Ayurveda, Siddha, Unani, and Chinese medicine [52]. Turmeric’s main active ingredient is curcumin, and its stability and bioavailability are the main issues, so it was modified into semi-synthetic analogs to make it more stable and potentiate its bioactivities [17]. Some of the curcumin analogues demonstrated superior antiproliferative activity than curcumin not only in the current study, but also in the previous study, where they demonstrated superior antiproliferative activity in single and five dose assays [18,19,20].

The molecular docking revealed that compound **3a** exhibited two π–π stacking with the *m*-chlorophenyl moiety with the residues Asp800 and Leu718, as well as one π–π stacking between one of the methoxyphenyl moiety and the residue Asp855. Compound **3a** demonstrated moderate antiproliferative activity with more than 50 percent sensitivity against MOLT-4 (% GI = 53.78), SR (% GI = 51.28), and HT29 (% GI = 53.43). The compound **3b** displayed two H-bonds with the phenolic functions with the residues Asp800 and Leu718, as well as two π–π stacking one between the methoxyphenyl moiety and the residue Asp855 and another with one of the hydrogen of doubly bonded carbon (C=C). Compound **3b** exhibited significant interaction within the active site of EGFR in molecular docking studies, and demonstrated better inhibition than curcumin against 35 of the 56 cell lines tested in common in single dose assay at 10 µM. Compound **3b** inhibited leukemia cancer panel better with GI_50_ values ranging from 1.52 to 3.38 µM, but the most promising inhibition with GI_50_ of 1.25 µM was observed against MDA-MB-435, while the least sensitivity was found against OVCAR-5 with GI_50_ value of 18.8 µM. Compound **3c** displayed three H-bonds with the phenolic functions with the residues Met793, Ser720, and Gln791, as well as four π–π stacking three between the *p*-trifluoromethylphenyl moiety and the residues Leu718 and Asp800 and another one with the pyrazoline CH and residue Asp855. The *p*-trifluoromethyl phenyl function lies within the hydrophobic cavity containing important residues Leu844, Cys797, and Met793. Compound **3c** inhibited leukemia cancer panel better with GI_50_ values ranging from 1.48 to 2.73 µM, and the most promising inhibition with GI_50_ of 1.25 µM was observed against SR, while the least inhibition was found against NCI-H226 with GI_50_ value of 7.29 µM. The SI values indicated that both the curcumin analogues (**3b**,**c**) were found to be non-selective against all the nine panels tested.

The curcumin analogs (**3a**–**c**) demonstrated promising antiproliferative activity and significant interaction within the active site of EGFR in molecular docking. Previous research has shown that curcumin has anti-EGFR activity [53]. Furthermore, many of the cancer cell lines, including breast, colon, non-small cell lung, renal, melanoma, ovarian, and prostate cancer cell lines, have been found to express varying levels of EGFR; thus, it was worth testing their anti-EGFR activity[30,31,32,33,34,35,36]. Compounds (**3a**–**c**) were found to be moderately inhibited EGFR. Compound **3a** had a higher docking score than compounds **3b**,**c**, but it showed moderate antiproliferative and anti-EGFR activities. Compounds **3b**,**c**, on the other hand, demonstrated significant antiproliferative and anti-EGFR activity, as we observed better interaction within the EGFR active site. Only π–π stacking interaction was observed with compound **3a**, while H-bond and π–π stacking interactions were observed with compounds **3b**,**c**.

In most of the cases, the chemical modifications of diketonic function of curcumin into pyrazole and primidinone analogues were found to be promising, whereas chemical modification of diketonic function that resulted in bigenelli-type curcumin compounds were found to be least significant [18,19,20,54]. Curcumin analogues previously reported in the literature demonstrated cytotoxicity on the CCGF-CEM cell line with IC_50_ values ranging from 3.13 to 93.40 µM, whereas compounds (**3b**,**c**) were found to be much more potent than the reported curcumin analogues [55].

## 5. Conclusions

Three curcumin analogs were prepared and tested for antiproliferative activity (**3a**–**c**). Compounds (**3a**–**c**) had docking scores >−6.337 kcal/mol, indicating efficient binding to the active site of EGFR. Compounds **3b**,**c** demonstrated promising antiproliferative activity in both one and five-dose assay. In a one-dose assay, compound **3b** demonstrated the most promising antiproliferative activity with mean % GI of 66.46, while compound **3c** demonstrated the most promising antiproliferative activity in five-dose assay with mean GI_50_ value of 2.84 µM, but none of the compounds were found to be selective against any panel in five-dose assay, as indicated by their selectivity ratio values. Except for compound **3a**, both compounds (**3b**,**c**) effectively inhibited the tested cancer cell lines in single and five dose assays. The curcumin analogues moderately inhibited the EGFR, a potential target for anticancer agents. Because the compounds demonstrated promising antiproliferative activity, the current report may lead to an expansion of the anticancer research development program in the future.

## Figures and Tables

**Table 1:** The molecular docking scores and interaction of ligands **3a**–**c**.

S. No.	Structure	Docking Score	Interaction
1	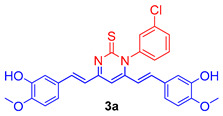	−6.593	π–π stacking (Asp855); π–π stacking (Asp800); π–π stacking (Leu718)
2	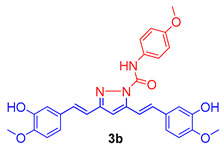	−6.337	H-bond (Leu718); H-bond (Asp800); π-π stacking (Leu718); π–π stacking (Asp800)
3	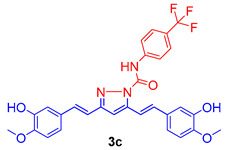	−6.452	H-bond (Met793); H-bond (Gln791); H-bond (Ser720); π–π stacking (Asp800); π–π stacking (Asp855); π–π stacking (Leu718)

**Table 2:** The antiproliferative activity of compounds **3a**–**c** in one dose (at 10 µM) and 5-dose assay (0.01 to 100 µM) against 60 NCI cancer cell lines.

Panel	Cell Line	GP and %GI at 10 µM						3b (NSC 782201)					3b (NSC 799011)				
		3a (NSC 799007)		3b (NSC 782201)		3c (NSC 799011)		GI_50_	Sub Panel MID ^b^	Selectivity Ratio (MID ^a^:MID ^b^)	TGI	LC_50_	GI_50_	Sub Panel MID ^b^	Selectivity Ratio (MID ^a^:MID ^b^)	TGI	LC_50_
		GP	%GI	GP	%GI	GP	%GI										
**Leukemia**	CCRF-CEM	53.43	46.57	**11.77**	**88.23**	**3.42**	**96.58**	2.34	2.59	1.89	32.7	>100	2.63	2.24	1.27	-	>100
	HL-60(TB)	83.48	16.52	**5.32**	**94.68**	**15.17**	**84.83**	2.55			6.69	>100	1.98			6.45	>100
	K-562	59.23	40.77	**9.25**	**90.75**	**9.45**	**90.55**	2.87			16.9	>100	2.53			>100	>100
	MOLT-4	**46.22**	**53.78**	**12.24**	**87.76**	**11.09**	**88.91**	2.87			13.3	>100	2.73			20.0	>100
	RPMI-8226	60.59	39.41	**21.30**	**78.70**	**−0.21**	**100.21**	3.38			29.2	>100	2.09			6.59	>100
	SR	**48.72**	**51.28**	-	-	**13.21**	**86.79**	1.52			12.8	>100	1.48			>100	>100
**Non-Small Cell Lung Cancer**	A549/ATCC	72.56	27.44	**27.29**	**72.71**	42.95	57.05	5.50	5.32	0.92	97.9	>100	4.11	3.44	0.82	>100	>100
	EKVX	85.81	14.19	52.39	47.61	**61.25**	**38.75**	6.62			83.7	>100	3.22			38.4	>100
	HOP-62	95.06	4.94	**32.75**	**67.25**	**62.87**	**37.13**	3.65			42.8	>100	3.60			16.2	>100
	HOP-92	-	-	89.07	10.93	-	-	7.77			>100	>100	2.35			6.79	>100
	NCI-H226	98.54	1.46	87.15	12.85	82.67	17.33	10.7			78.6	>100	7.29			27.6	91.7
	NCI-H23	90.95	9.05	**43.94**	**56.06**	51.29	48.71	3.81			63.8	>100	2.13			6.48	>100
	NCI-H322M	91.93	8.07	58.14	41.86	82.70	17.30	3.87			72.5	>100	3.25			12.9	49.6
	NCI-H460	89.51	10.49	**14.22**	**85.78**	**18.29**	**81.71**	4.24			31.4	>100	3.22			9.95	50.7
	NCI-H522	37.89	62.11	**−13.52**	**113.52**	**7.32**	**92.68**	1.76			12.7	>100	1.78			4.65	40.8
**Colon Cancer**	COLO 205	107.08	−7.08	106.58	−6.58	51.27	48.73	16.2	5.77	0.85	44.3	>100	2.05	2.17	1.31	4.50	9.91
	HCC-2998	103.70	−3.70	74.91	25.09	102.63	−2.63	5.78			>100	>100	2.04			3.82	71.2
	HCT-116	61.88	38.12	**9.13**	**90.87**	**−53.67**	**153.67**	3.22			42.9	>100	1.51			2.94	5.70
	HCT-15	93.93	6.07	**13.17**	**86.87**	**25.51**	**74.49**	2.98			>100	>100	2.69			9.63	>100
	HT29	**46.57**	**53.43**	**31.26**	**68.74**	**3.50**	**96.50**	3.52			11.0	>100	2.02			4.13	8.44
	KM12	80.33	19.67	**17.89**	**82.11**	**41.38**	**58.62**	3.19			20.7	>100	2.33			5.59	54.0
	SW-620	89.48	10.52	**27.10**	**72.90**	**18.00**	**82.00**	5.51			61.6	>100	2.56			5.90	33.6
**CNS Cancer**	SF-268	83.76	16.24	**41.81**	**58.19**	**37.12**	**62.88**	3.21	3.83	1.28	36.4	>100	2.80	2.78	1.02	15.4	>100
	SF-295	109.94	−9.94	51.62	48.38	83.42	16.58	6.25			53.2	>100	2.89			9.85	76.5
	SF-539	93.11	6.89	**6.72**	**93.28**	**29.01**	**70.99**	2.35			13.6	>100	2.37			7.45	56.0
	SNB-19	84.55	15.45	**38.85**	**61.15**	**48.68**	**51.32**	5.28			86.7	>100	3.16			11.2	36.9
	SNB-75	80.78	19.22	**32.61**	**67.39**	71.80	28.20	2.35			16.3	>100	2.99			66.0	>100
	U251	59.41	40.59	**26.03**	**73.97**	**9.71**	**90.29**	3.55			20.9	>100	2.49			6.86	>100
**Melanoma**	LOX IMVI	78.64	21.36	**27.59**	**72.41**	**9.11**	**90.89**	3.95	4.59	1.06	>100	>100	1.77	2.51	1.31	3.71	-
	MALME-3M	89.46	10.54	51.86	48.14	52.27	47.73	3.66			38.6	>100	2.89			8.40	32.6
	M14	94.04	5.96	**4.97**	**95.03**	**29.48**	**70.52**	3.15			41.5	>100	1.94			4.08	8.58
	MDA-MB-435	81.32	18.68	**−36.25**	**136.25**	**27.85**	**72.15**	1.25			4.68	>100	2.35			7.06	33.4
	SK-MEL-2	95.15	4.85	**21.38**	**78.62**	84.47	15.53	3.23			18.1	>100	3.15			17.3	>100
	SK-MEL-28	92.44	7.56	**34.53**	**65.47**	57.99	42.01	3.52			92.7	>100	2.19			5.90	23.9
	SK-MEL-5	78.99	21.01	**9.04**	**90.96**	**35.40**	**64.60**	3.67			16.1	>100	2.27			6.89	28.0
	UACC-257	52.91	47.09	75.22	24.78	**25.01**	**74.99**	16.7			64.8	>100	4.01			14.0	42.0
	UACC-62	82.19	17.81	**25.47**	**74.53**	**31.10**	**68.90**	2.14			6.91	>100	2.02			4.76	16.7
**Ovarian Cancer**	IGROV1	95.26	4.74	**34.39**	**65.61**	**42.82**	**57.18**	4.49	6.46	0.76	57.0	>100	2.77	3.24	0.88	11.6	>100
	OVCAR-3	91.30	8.70	**13.62**	**86.38**	**13.76**	**86.24**	2.98			8.40	>100	3.41			26.7	>100
	OVCAR-4	82.55	17.45	51.51	48.49	76.71	23.29	5.72			90.7	>100	3.51			52.7	>100
	OVCAR-5	112.92	−12.92	62.30	37.70	115.18	−15.16	18.8			54.1	>100	2.16			4.97	15.2
	OVCAR-8	71.43	28.57	32.79	67.21	**0.43**	**99.57**	5.32			59.9	>100	3.33			>100	>100
	NCI/ADR-RES	96.37	3.63	**23.89**	**76.11**	51.65	48.35	4.28			>100	>100	3.51			>100	>100
	SK-OV-3	100.88	−0.88	60.74	39.26	66.09	33.91	3.66			27.5	>100	4.02			66.7	>100
**Renal Cancer**	786–0	94.62	5.38	**32.08**	**67.92**	**20.07**	**79.93**	4.00	5.84	0.84	24.3	>100	2.42	3.06	0.93	7.52	>100
	A498	104.38	−4.38	82.47	17.53	108.03	-8.03	17.2			56.4	>100	5.81			2.06	57.3
	ACHN	95.01	4.99	**26.03**	**77.93**	65.82	34.19	4.18			>100	>100	2.48			7.08	>100
	CAKI-1	79.04	20.96	**38.06**	**61.94**	54.99	45.01	3.60			>100	>100	3.02			12.7	44.1
	RXF 393	92.63	7.37	**23.01**	**76.99**	**22.66**	**77.34**	3.02			8.87	>100	1.98			3.73	-
	SN 12C	92.61	7.39	**31.45**	**68.55**	**36.92**	**63.08**	3.05			33.7	>100	2.73			9.90	>100
	TK-10	112.60	−12.60	63.84	36.16	77.69	22.31	10.2			95.3	>100	4.13			14.8	59.4
	UO-31	71.26	28.74	**22.67**	**77.33**	**42.85**	**57.15**	1.52			36.4	>100	1.94			-	>100
**Prostate Cancer**	PC-3	89.21	10.79	**28.43**	**71.57**	**38.03**	**61.97**	5.11	4.55	1.07	>100	>100	3.21	3.44	0.82	13.5	>100
	DU-145	80.84	19.16	**27.23**	**72.77**	**47.55**	**52.45**	3.99			24.6	>100	3.67			13.0	45.3
**Breast Cancer**	MCF7	67.91	32.09	**15.63**	**84.37**	**22.92**	**77.08**	3.30	4.12	1.19	17.0	>100	1.97	2.86	0.99	10.7	72.0
	MDA-MB-231	68.25	31.75	**36.53**	**63.47**	54.79	45.21	4.94			83.5	>100	3.06			9.62	>100
	HS 578T	84.33	15.67	**28.71**	**71.29**	51.89	48.11	6.77			61.9	>100	5.10			60.6	>100
	BT-549	72.52	27.48	**31.37**	**68.63**	**18.97**	**81.03**	2.49			29.5	>100	1.96			3.67	6.86
	T-47D	63.82	36.18	**34.83**	**65.17**	**47.40**	**52.60**	3.68			25.8	>100	2.81			39.0	>100
	MDA-MB-468	83.81	16.19	**34.68**	**65.32**	**43.79**	**56.21**	3.55			21.6	>100	2.30			6.25	>100
Mean		82.32	17.68	**33.54**	**66.46**	**40.74**	**59.26**										
Total cell line and sum of concentration	60							293.96					170.18				
MID ^a^								4.89					2.84				

% GI = 100–GP; (-) = Not tested; Black font =< 50% GI; Bold & blue font = GIs > 50 (GPs < 50); Bold & green font = GIs > 68 (GPs < 32); Bold & red font = GIs >100 (GPs = < 0 means lethal effect) (One-dose assay); NSC Number: Originally known as Cancer Chemotherapy National Service Center number, and it is an identifying number assigned by Developmental therapeutics Program (DTP) to an agent or product (e.g., small molecule or biological agent); MID ^a^ and MID ^b^ were the mean GI_50_ of 60 NCI cancer cell lines and the individual cancer cell line panels; Selectivity ration = MID^a^/MID^b^; GI_50_, LC_50_ and TGI stand for 50% growth inhibition, 50% lethal concentration, and total growth inhibition respectively.

**Table 3:** Mean % GI of compounds **3a**–**c**, curcumin and gefitinib at 10 µM.

Panel	3a	3b	3c	Cur *	Gefitinib ^#^
Leukemia	41.39	88.02	91.31	**97.76**	79.68
Non-Small cell lung cancer	17.22	56.51	48.83	49.27	**63.97**
Colon Cancer	16.72	60	73.05	**95.76**	52.19
CNS Cancer	14.74	**67.06**	53.38	60.75	46.13
Melanoma	17.21	**76.24**	60.81	54.63	44.99
Ovarian Cancer	7.04	60.11	47.62	44.66	**60.93**
Renal Cancer	7.23	60.54	46.37	45.35	**77.89**
Prostate Cancer	14.98	**72.17**	57.21	61.3	59.6
Breast Cancer	26.56	**69.71**	60.04	56.1	52.88

* Cur = Curcumin; The anticancer activity of curcumin was retrieved from NCI data-ware with NSC ID 32982 [43]; Bold font represents the best result; ^#^ The anticancer activity of gefitinib was retrieved from NCI data-ware with NSC ID 759856 [43].

## Data Availability

The authors confirm that the data supporting the study’s findings are included in the article and its supplementary information.

## Supplementary Materials

The following are available online at https://www.mdpi.com/article/10.3390/plants10081559/s1, Figure S1: Antiproliferative profile of compound **3b**, Figures S2–S4: Anticancer data of compounds **3a-c** in single dose assay.
