# Supplementary material for: Molecular Engineering of Curcumin, an Active Constituent of Curcuma longa L. (Turmeric) of the Family Zingiberaceae with Improved Antiproliferative Activity
Source: Plants (Basel). 2021 Jul 29;10(8):1559. doi: 10.3390/plants10081559 (PMC8398451; doi:10.3390/plants10081559)
Supplement: Supplementary file 1 [file plants-10-01559-s001.zip › plants-1286660-supplementary.pdf]

Article

Molecular Engineering of Curcumin, an Active Constituent of *Curcuma longa* L. (Turmeric) of the Family *Zingiberaceae* with Improved Antiproliferative Activity

Amena Ali [1,*,†], Abuzer Ali [2,†], Abu Tahir [3], Md. Afroz Bakht [4], Salahuddin [5] and Mohamed Jawed Ahsan [6,*]

[1] Department of Pharmaceutical Chemistry, College of Pharmacy, Taif University, P.O. Box 11099, Taif 21944, Saudi Arabia
[2] Department of Pharmacognosy, College of Pharmacy, Taif University, P.O. Box 11099, Taif 21944,
Saudi Arabia; abuali@tu.edu.sa
[3] Department of Pharmacology, Raghukul College of Pharmacy, Bhopal, Madhya Pradesh 462 003, India; aliabutahir2009@gmail.com
[4] Department of Chemistry, College of Science and Humanity Studies, Prince Sattam Bin Abdulaziz
University, P.O. Box83, Al-Kharj 11942, Saudi Arabia; bakhtpharm@gmail.com
[5] Department of Chemistry, Noida Institute of Technology (Pharmacy Institute), Knowledge Park-2,
Greater Noida, Uttar Pradesh 201 306, India; sallu_05@yahoo.co.in
[6] Department of Pharmaceutical Chemistry, Maharishi Arvind College of Pharmacy, Ambabari Circle, Jaipur, Rajasthan 302 039, India
* Correspondence: amrathore@tu.edu.sa (A.A.); jawedpharma@gmail.com (M.J.A.)
† Authors contributed equally.

Supplementary Information

Antiproliferative activity

The anticancer screening was carried out as per the NCI US protocol [1-5]. Using the seven absorbance measurements (time zero, (Ti), control growth, (C), and test growth in the presence of drug at the five concentration levels (Tf)), the percentage growth was calculated at each of the drug concentrations level as [($\frac{Tf-Ti}{C-Ti} \times 100$ for concentrations for which $T_f \geq T_i$, and $\frac{Tf-Ti}{Ti} \times 100$ for concentrations for which $T_f < T_i$.

Three-dose response parameters (GI_{50}, TGI, and LC_{50}) were calculated for each of the experimental agents. Growth inhibition of 50% (GI_{50}) was calculated from $100 \times \frac{Tf-Ti}{C-Ti} = 50$, , which was the drug concentration resulting in a 50% reduction in the net protein increase (as measured by sulforhodamine B, SRB staining) in control cells during the drug incubation. The total growth inhibition (TGI) was calculated from $T_f = T_i$, which was the drug concentration resulting in total growth inhibition and signified the cytostatic effect. The LC_{50} was calculated from $100 \times \frac{Tf-Ti}{C-Ti} = -50$, , indicating a net loss of cells following treatment which indicated the concentration of drug resulting in a 50% reduction in the measured protein at the end of the drug treatment as compared to that at the beginning.

References

1. DTP Developmental therapeutic Programs: http://dtp.nci.nih.gov

2. Monks, A.; Scudiero, D.; Skehan, P.; Shoemaker, R.; Paull, K.; Vistica, D.; Hose, C.; Langley, J.; Cronise, P.; Vaigro-Wolff, A.; Gray-Goodrich, M.; Campbell, H.; Mayo, J.; Boyd, M. Feasibility of a highflux anticancer drug screening using a diverse panel of cultured human tumor cell lines. J. Nat. Cancer Inst. 1991, 83 757-766. https://doi.org/10.1093/jnci/83.11.757

3. Boyd, M.R.; Paull, K.D. Some practical considerations and applications of the National Cancer Institute in vitro anticancer drug discovery screen. Drug Dev. Res. 1995, 34, 91-109. https://doi.org/10.1002/ddr.430340203

4. Shoemaker, R.H. The NCI60 human tumour cell line anticancer drug screen. Nat. Rev. Cancer. 2006, 6, 813-823. https://doi.org/10.1038/nrc1951

5. Grever, M. R.; Schepartz, S. A.; Chabner, B. A. Sem. Oncol. 1992, 19, 622–638.

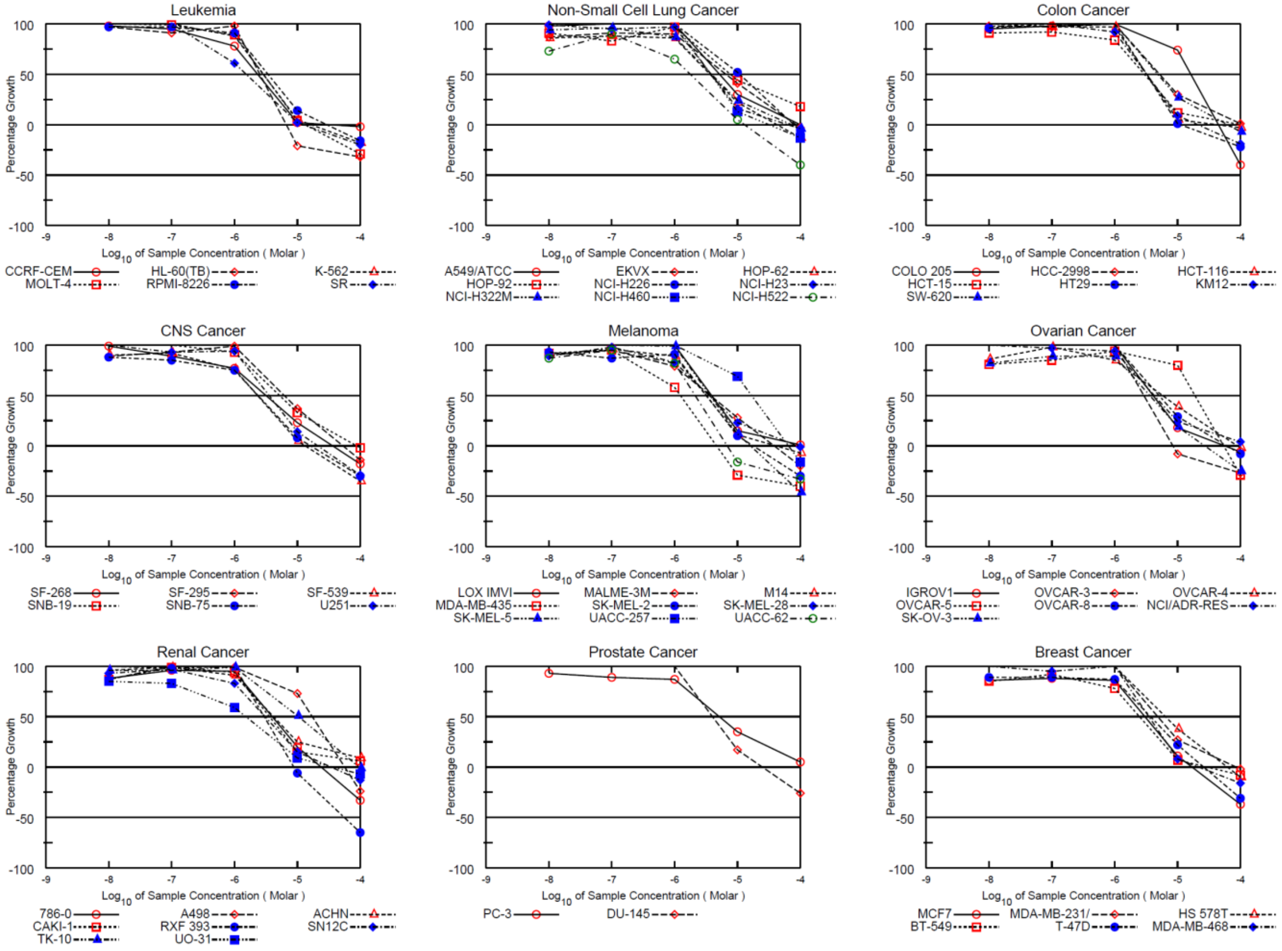

Figure S1. Antiproliferative profile of compound **3b** in terms of GP and Log_{10} molar concentration in five dose assay.

Developmental Therapeutics Program One Dose Mean Graph	NSC: D-799007 / 1	Conc: 1.00E-5 Molar	Test Date: Jul 10, 2017
	Experiment ID: 1707OS32		Report Date: Aug 03, 2017

Panel/Cell Line	Growth Percent
Leukemia	
CCRF-CEM	53.43
HL-60(TB)	83.48
K-562	59.23
MOLT-4	46.22
RPMI-8226	60.59
SR	48.72
Non-Small Cell Lung Cancer	
A549/ATCC	72.56
EKVX	85.81
HOP-62	95.06
NCI-H226	98.54
NCI-H23	90.95
NCI-H322M	91.93
NCI-H460	89.51
NCI-H522	37.89
Colon Cancer	
COLO 205	107.08
HCC-2998	103.70
HCT-116	61.88
HCT-15	93.93
HT29	46.57
KM12	80.33
SW-620	89.48
CNS Cancer	
SF-268	83.76
SF-295	109.94
SF-539	93.11
SNB-19	84.55
SNB-75	80.78
U251	59.41
Melanoma	
LOX IMVI	78.64
MALME-3M	89.46
M14	94.04
MDA-MB-435	81.32
SK-MEL-2	95.15
SK-MEL-28	92.44
SK-MEL-5	78.99
UACC-257	52.91
UACC-62	82.19
Ovarian Cancer	
IGROV1	95.26
OVCAR-3	91.30
OVCAR-4	82.55
OVCAR-5	112.92
OVCAR-8	71.43
NCI/ADR-RES	96.37
SK-OV-3	100.88
Renal Cancer	
786-0	94.62
A498	104.38
ACHN	95.01
CAKI-1	79.04
RXF 393	92.63
SN12C	92.61
TK-10	112.60
UO-31	71.26
Prostate Cancer	
PC-3	89.21
DU-145	80.84
Breast Cancer	
MCF7	67.91
MDA-MB-231/ATCC	68.25
HS 578T	84.33
BT-549	72.52
T-47D	63.82
MDA-MB-468	83.81
Mean	82.32
Delta	44.43
Range	75.03

Mean Growth Percent - Growth Percent

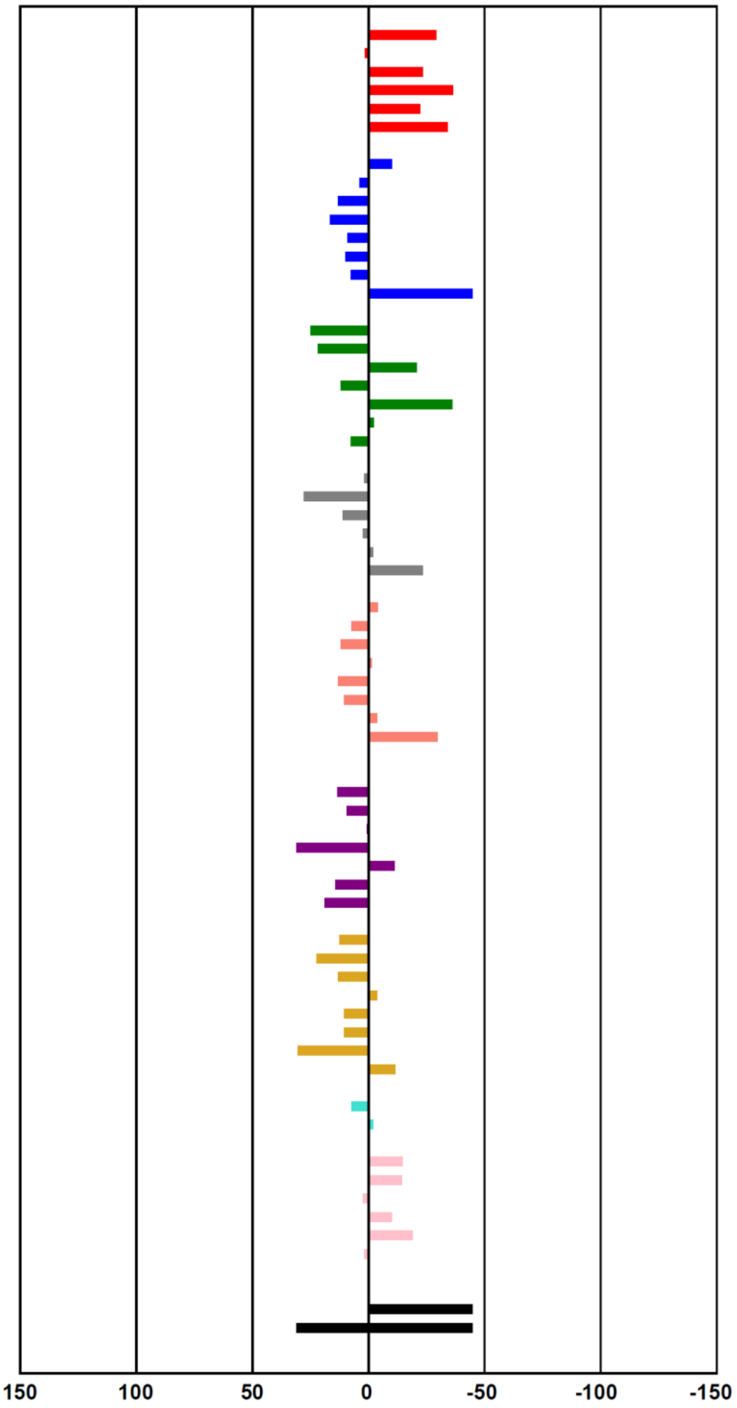

Figure S2. 60 NCI cancer cell lines based antiproliferative activity of curcumin analogue **3a** in single dose assay at 10 μM.

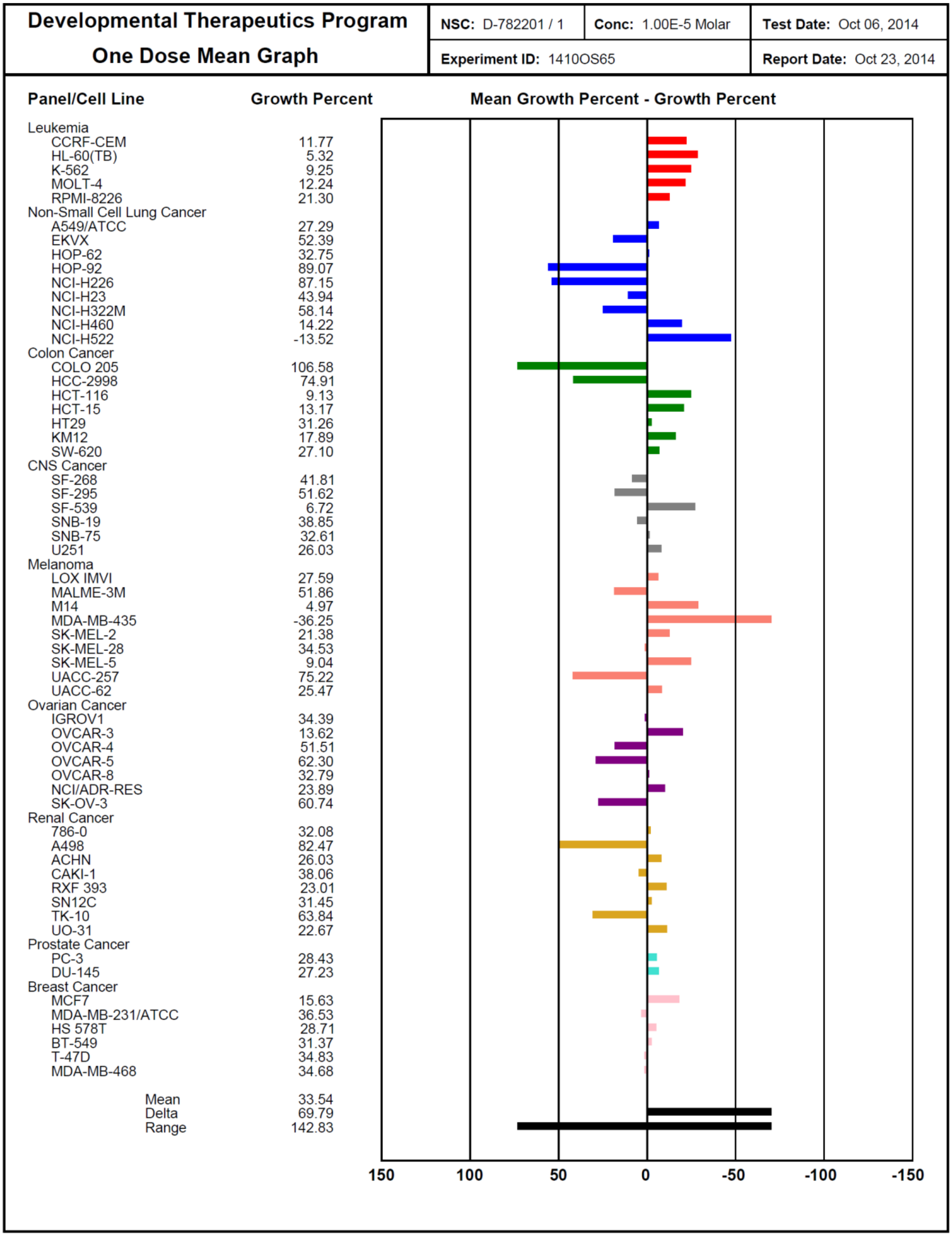

Figure S3. 60 NCI cancer cell lines based antiproliferative activity of curcumin analogue **3b** in single dose assay at 10 μM.

Developmental Therapeutics Program One Dose Mean Graph	NSC: D-799011 / 1	Conc: 1.00E-5 Molar	Test Date: Jul 10, 2017
	Experiment ID: 1707OS32		Report Date: Aug 03, 2017

Panel/Cell Line	Growth Percent
**Leukemia**	
CCRF-CEM	3.42
HL-60(TB)	15.17
K-562	9.45
MOLT-4	11.09
RPMI-8226	-0.21
SR	13.21
**Non-Small Cell Lung Cancer**	
A549/ATCC	42.95
EKVX	61.25
HOP-62	62.87
NCI-H226	82.67
NCI-H23	51.29
NCI-H322M	82.70
NCI-H460	18.29
NCI-H522	7.32
**Colon Cancer**	
COLO 205	51.27
HCC-2998	102.63
HCT-116	-53.67
HCT-15	25.51
HT29	3.50
KM12	41.38
SW-620	18.00
**CNS Cancer**	
SF-268	37.12
SF-295	83.42
SF-539	29.01
SNB-19	48.68
SNB-75	71.80
U251	9.71
**Melanoma**	
LOX IMVI	9.11
MALME-3M	52.27
M14	29.48
MDA-MB-435	27.85
SK-MEL-2	84.47
SK-MEL-28	57.99
SK-MEL-5	35.40
UACC-257	25.01
UACC-62	31.10
**Ovarian Cancer**	
IGROV1	42.82
OVCAR-3	13.76
OVCAR-4	76.71
OVCAR-5	115.18
OVCAR-8	0.43
NCI/ADR-RES	51.65
SK-OV-3	66.09
**Renal Cancer**	
786-0	20.07
A498	108.03
ACHN	65.82
CAKI-1	54.99
RXF 393	22.66
SN12C	36.92
TK-10	77.69
UO-31	42.85
**Prostate Cancer**	
PC-3	38.03
DU-145	47.55
**Breast Cancer**	
MCF7	22.92
MDA-MB-231/ATCC	54.79
HS 578T	51.89
BT-549	18.97
T-47D	47.40
MDA-MB-468	43.79
Mean	40.74
Delta	94.41
Range	168.85

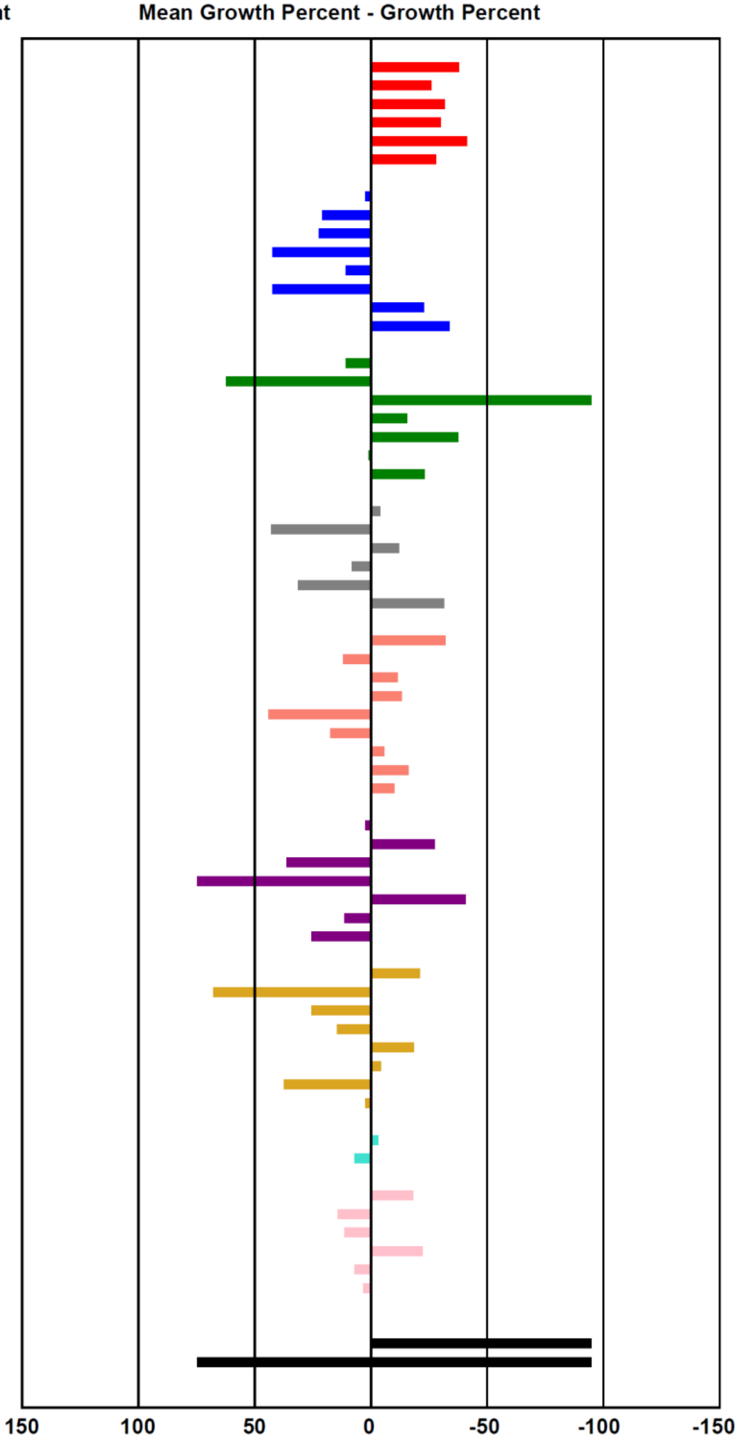

Figure S4. 60 NCI cancer cell lines based antiproliferative activity of curcumin analogue **3c** in single dose assay at 10 μM.
